# Association of Parental Health Literacy with Treatment Adherence and Clinical Outcomes in Adolescents with Allergic Rhinitis: A Controlled Longitudinal Study

**DOI:** 10.3390/jcm15176658

**Published:** 2026-08-28

**Authors:** Fatih Kaplan, Abdulgani Gülyüz, Bilge Kurnaz Kaplan

**Affiliations:** 1 Department of Pediatric Allergy and Immunology, Malatya Training and Research Hospital, Malatya 44000, Türkiye; 2 Department of Pediatrics, Faculty of Medicine, Malatya Turgut Özal University, Malatya 44210, Türkiye; 3 Department of Otorhinolaryngology (ENT), Malatya Training and Research Hospital, Malatya 44090, Türkiye

**Keywords:** allergic rhinitis, adolescents, health literacy, parental health literacy, treatment adherence, Pediatric Sleep Questionnaire, sleep-related symptoms

## Abstract

**Background**: Parental health literacy may be associated with treatment adherence and clinical outcomes in adolescents with allergic rhinitis (AR), but evidence regarding these relationships remains limited. Objective: To evaluate the associations of parental health literacy with treatment adherence and clinical outcomes in adolescents with AR and to compare parental health literacy and baseline sleep-related symptom burden between adolescents with AR and healthy controls. **Methods**: In this controlled longitudinal observational study, 132 adolescents with AR and 120 healthy controls were enrolled. Parental health literacy was assessed using the Turkish Health Literacy Scale-32 (TSOY-32). Adolescents with AR were evaluated at baseline (T0), week 4 (T1), and week 8 (T2). Nasal symptoms were assessed using the Total Nasal Symptom Score (TNSS) and Visual Analog Scale (VAS), and sleep-related symptoms using the Pediatric Sleep Questionnaire (PSQ). Treatment adherence was classified at T2 as a single binary measure covering the entire 8-week follow-up period. Multivariable linear regression and Firth penalized logistic regression analyses were performed. **Results**: Adolescents with AR had significantly higher PSQ total and subscale scores and a greater prevalence of PSQ-defined high risk than healthy controls (all *p* < 0.001). During follow-up, PSQ, TNSS, and VAS scores decreased significantly (all *p* < 0.001), with greater improvements among treatment-adherent adolescents. Parental health literacy was associated with treatment adherence (*p* < 0.001) and with improvements in TNSS and VAS. However, after adjustment, treatment adherence—but not parental health literacy—remained independently associated with T2 PSQ, TNSS, and VAS scores. The multivariable adherence model showed high apparent discrimination within the study sample (AUC = 0.969). **Conclusions**: Higher treatment adherence was consistently associated with more favorable clinical outcomes in adolescents with AR. Parental health literacy was associated with treatment adherence but was not independently associated with T2 clinical outcomes after multivariable adjustment. Whether treatment adherence represents a pathway linking parental health literacy to clinical outcomes cannot be determined from the present observational study.

## 1. Introduction

Allergic rhinitis (AR) is one of the most common chronic inflammatory diseases in childhood and adolescence and represents a substantial public health burden because of its adverse associations with quality of life, academic performance, and daily functioning. Its prevalence among children and adolescents has been estimated to range from 10% to 30%. Beyond the characteristic symptoms of nasal congestion, rhinorrhea, sneezing, and nasal itching, AR is also associated with sleep-related problems, including snoring, daytime fatigue, impaired concentration, and behavioral symptoms, which may further contribute to disease burden and functional impairment [1,2,3,4,5,6].

Effective management of AR involves not only appropriate pharmacological treatment but also consistent implementation of treatment recommendations by adolescents and their caregivers. Treatment adherence is particularly relevant for controller therapies such as intranasal corticosteroids, and poor adherence has been associated with inadequate symptom control, impaired quality of life, and increased healthcare utilization [7,8,9]. In pediatric chronic disease management, treatment-related behaviors are also shaped by the family environment because parents or primary caregivers frequently participate in medication administration, treatment supervision, and healthcare follow-up.

Health literacy refers to an individual’s ability to access, understand, evaluate, and appropriately use health-related information. Parental health literacy may therefore be particularly relevant in adolescents with chronic conditions, as it may be associated with caregivers’ understanding of treatment instructions, management of prescribed therapies, and engagement with healthcare services. Previous studies have linked parental health literacy to a range of treatment-related behaviors and child health outcomes; however, evidence remains limited regarding how parental health literacy and treatment adherence are concurrently associated with longitudinal changes in both nasal and sleep-related symptoms among adolescents with AR [10,11,12,13].

This question is clinically relevant because sleep-related symptoms constitute an important component of AR morbidity and can be assessed using validated screening instruments such as the Pediatric Sleep Questionnaire (PSQ) [14,15,16].

Therefore, the present controlled longitudinal observational study aimed to evaluate the associations of parental health literacy with treatment adherence and repeated nasal and sleep-related clinical outcomes in adolescents with AR. We additionally aimed to compare parental health literacy and baseline sleep-related symptom burden between adolescents with AR and healthy controls.

## 2. Materials and Methods

### 2.1. Study Design and Participants

This controlled longitudinal observational study was conducted between February and June 2026 in the Departments of Pediatrics and Otorhinolaryngology at Malatya Training and Research Hospital. The study was designed to evaluate the associations of parental health literacy with treatment adherence and longitudinal clinical outcomes in adolescents with isolated allergic rhinitis (AR), and to compare parental health literacy and baseline sleep-related symptoms between adolescents with AR and healthy controls. Adolescents with AR were evaluated at baseline (T0), week 4 (T1), and week 8 (T2), whereas healthy controls underwent baseline assessment only.

Adolescents aged 12–18 years attending the participating outpatient clinics during the study period were screened for eligibility. The AR group included adolescents with isolated AR diagnosed according to the Allergic Rhinitis and its Impact on Asthma (ARIA) criteria [7]. The control group comprised consecutive healthy adolescents attending the same pediatric and otorhinolaryngology outpatient clinics during the study period who had no diagnosis of AR or any other chronic allergic disease. No individual or frequency matching was performed between the AR and control groups.

For the AR group, adolescents with physician-diagnosed asthma were excluded to maintain an isolated AR population. Individuals with incomplete clinical data or study questionnaires, as well as those who declined participation, were excluded. In the control group, adolescents with chronic systemic diseases or a previously diagnosed sleep-related breathing disorder were excluded. Parents or primary caregivers with cognitive or communication impairments that prevented independent completion of the Turkish Health Literacy Scale-32 (TSOY-32) were also excluded.

The study was approved by the Clinical Research Ethics Committee of Malatya Turgut Özal University Faculty of Medicine (Approval No. 2026/16; 14 January 2026) and was conducted in accordance with the Declaration of Helsinki. Written informed consent was obtained from all parents or legal guardians, and assent was obtained from adolescents when appropriate according to age.

### 2.2. Data Collection

Demographic and clinical data for both adolescents and their parents were collected using a standardized data collection form. Information regarding age, sex, family history of atopy, coexisting allergic diseases, prescribed treatment, treatment adherence, and clinical findings was obtained from medical records, the electronic medical record system, and outpatient clinic evaluations. Parental health literacy was assessed using the Turkish Health Literacy Scale-32 (TSOY-32).

Adolescents with AR underwent clinical evaluation at baseline (T0), week 4 (T1), and week 8 (T2). At each assessment, nasal and sleep-related clinical outcomes were evaluated using the predefined study instruments. Treatment adherence was not classified separately at each visit; instead, a single overall adherence classification covering the complete 8-week follow-up period was assigned at T2, as described below.

### 2.3. Clinical Assessment

Nasal symptom severity was evaluated using the Total Nasal Symptom Score (TNSS) and the Visual Analog Scale (VAS). AR persistence and severity were classified according to the ARIA guidelines as intermittent or persistent and as mild or moderate-to-severe, respectively [7,17,18].

Sleep-related breathing symptoms were assessed at T0, T1, and T2 using the Pediatric Sleep Questionnaire (PSQ), completed by the same parent or primary caregiver whenever possible. The total PSQ score was calculated according to the recommended scoring method, with a score of ≥0.33 considered indicative of high risk for a sleep-related breathing disorder. The PSQ was used solely as a screening instrument and was not intended to establish a diagnosis of obstructive sleep apnea [16].

For longitudinal analyses, improvement scores were calculated as the baseline value minus the corresponding follow-up value (T0−T1 and T0−T2), such that positive values indicated clinical improvement.

### 2.4. Assessment of Parental Health Literacy

Parental health literacy was assessed once at baseline (T0) using the Turkish Health Literacy Scale-32 (TSOY-32). The questionnaire was completed independently by a parent or primary caregiver for each adolescent. Whenever possible, it was completed by the caregiver primarily responsible for the adolescent’s daily treatment and healthcare follow-up [19].

Health literacy was classified according to the established TSOY-32 categories as inadequate, problematic/limited, adequate, and excellent. The analytical dataset retained these predefined four categories rather than the original continuous total score; therefore, parental health literacy was analyzed categorically. For ordinal analyses, the categories were coded in their natural ordered sequence from inadequate to excellent, whereas in the primary Firth penalized logistic regression model they were entered as nominal categories, with inadequate health literacy serving as the reference category [19].

### 2.5. Assessment of Treatment Adherence

Treatment adherence was classified by the evaluating physician at the T2 assessment, taking the participant’s overall adherence throughout the entire 8-week follow-up period into consideration. A single binary classification (adherent vs. non-adherent) was assigned for the complete follow-up period; separate adherence classifications were not generated at T1 and T2.

The classification was based on reports from both the adolescent and the parent regarding adherence to the prescribed medication dose, frequency, and duration, together with attendance at scheduled follow-up visits. No prespecified quantitative adherence threshold or validated adherence scale was used. Adherence was not objectively verified using electronic medication monitoring, pill counts, or prescription-refill records.

In cases of discordance between adolescent and parent reports, the final classification was determined by the evaluating physician based on the perceived consistency and credibility of the information provided during clinical follow-up. Medication-taking behavior and attendance at scheduled follow-up visits were incorporated into the same composite adherence assessment and were not retained as separate variables in the analytical dataset.

At the time of adherence classification, the physician assessing treatment adherence had access to the TNSS and VAS findings but was blinded to the PSQ results. Accordingly, adherence assessment was not blinded to nasal symptom outcomes but was blinded to the PSQ outcome.

### 2.6. Statistical Analysis

Statistical analyses were performed using IBM SPSS Statistics for Windows, Version 26.0 (IBM Corp., Armonk, NY, USA). Firth penalized logistic regression analyses were conducted in R version 4.5.1 (R Foundation for Statistical Computing, Vienna, Austria) using the logistf package (version 1.26.1). Data distribution was assessed using histograms, Q–Q plots, and the Shapiro–Wilk test. Continuous variables with non-normal distributions are presented as medians and interquartile ranges (IQRs), whereas categorical variables are presented as frequencies and percentages.

Comparisons between two independent groups were performed using the Mann–Whitney U test for continuous variables and the Pearson χ^2^ test or Fisher’s exact test, as appropriate, for categorical variables. Changes in PSQ, TNSS, and VAS scores across T0, T1, and T2 within the allergic rhinitis group were analyzed using the Friedman test. When the overall test was significant, pairwise comparisons were performed using Bonferroni-adjusted Wilcoxon signed-rank tests. Changes in the proportion of participants classified as high risk according to the PSQ between T0–T1 and T0–T2 were evaluated using Bonferroni-adjusted McNemar tests.

Clinical improvement scores were compared across parental health literacy categories using the Kruskal–Wallis test. The ordinal association between parental health literacy and clinical improvement was assessed using Spearman’s rank correlation analysis.

Separate multivariable linear regression models were constructed to examine factors associated with T2 PSQ, TNSS, and VAS scores. Each model included the corresponding baseline score, treatment adherence, treatment step, age, sex, allergic rhinitis severity, and parental TSOY-32 category as covariates. HC3 heteroscedasticity-consistent standard errors were applied in all linear regression models. Multicollinearity was evaluated using variance inflation factors.

Factors associated with treatment adherence were examined using Firth penalized multivariable logistic regression because of sparse category-specific cells and the potential for separation. In the primary model, parental TSOY-32 categories were entered as nominal categorical variables, with inadequate health literacy as the reference category. Age, treatment step, and baseline PSQ total score were also included as covariates. Odds ratios for the baseline PSQ score were reported per 0.10-point increase. The overall contribution of parental TSOY-32 categories was evaluated using the penalized likelihood ratio test.

As a sensitivity analysis to assess the robustness of the association in the presence of sparse category-specific cells, the four TSOY-32 categories were additionally entered as a single ordinal term. This analysis was intended to assess the consistency of the direction of association under a more parsimonious model specification rather than to replace the primary categorical model.

Model discrimination was summarized using the area under the receiver operating characteristic curve (AUC). Because the AUC was calculated from predictions generated in the same dataset used to fit the model and no internal or external validation was performed, it was interpreted as apparent, in-sample discrimination rather than validated predictive performance. All statistical tests were two-sided, and a *p* value < 0.05 was considered statistically significant.

## 3. Results

### 3.1. Study Population and Baseline Characteristics

A total of 252 adolescents were included in the study, comprising 132 patients with allergic rhinitis and 120 healthy controls. There were no significant differences between the groups in terms of age [15.00 (13.00–16.00) vs. 15.00 (14.00–16.00) years, respectively; *p* = 0.514] or the proportion of males (48.5% vs. 49.2%; *p* = 0.914).

At baseline, the allergic rhinitis group had significantly higher PSQ total scores as well as snoring, sleepiness, and behavior subscale scores than the control group (all *p* < 0.001). Likewise, the proportion of participants classified as high risk according to the PSQ was significantly greater in the allergic rhinitis group than in the control group (50.0% [*n* = 66] vs. 5.0% [*n* = 6], *p* < 0.001).

Among parents of adolescents with allergic rhinitis, 19.7% had inadequate, 28.0% problematic/limited, 36.4% adequate, and 15.9% excellent health literacy. The corresponding proportions in the control group were 25.0%, 28.3%, 32.5%, and 14.2%, respectively. The distribution of parental health literacy categories did not differ significantly between the two groups (*p* = 0.754) (Table 1).

### 3.2. Changes in Clinical Outcomes During Follow-Up

Among adolescents with allergic rhinitis, significant changes were observed in PSQ total and subscale scores, as well as TNSS and VAS scores, across the three assessment time points (T0, T1, and T2) (all Friedman tests, *p* < 0.001).

The median PSQ total score decreased from 0.32 (0.00–0.42) at T0 to 0.12 (0.00–0.35) at T1 and 0.00 (0.00–0.17) at T2. The median TNSS decreased from 7.00 (5.00–9.00) at baseline to 3.00 (2.00–6.00) at both T1 and T2. Similarly, the median VAS score declined from 6.00 (5.00–8.00) at baseline to 3.00 (2.00–5.00) at both follow-up visits.

The proportion of participants classified as high risk according to the PSQ decreased from 50.0% (*n* = 66) at baseline to 28.0% (*n* = 37) at T1 and 19.7% (*n* = 26) at T2. Compared with baseline, the reduction was significant at both follow-up assessments (Bonferroni-adjusted McNemar test; both *p* < 0.001) (Table 2).

### 3.3. Treatment Adherence and Clinical Outcomes

Among the 132 adolescents with allergic rhinitis, 90 (68.2%) were classified as treatment-adherent and 42 (31.8%) as non-adherent. Treatment-adherent adolescents were older than non-adherent participants [15.00 (14.00–16.00) vs. 14.00 (13.00–15.75) years, *p* = 0.041]. They also had lower baseline PSQ, TNSS, and VAS scores, as well as lower treatment step levels (all *p* ≤ 0.001). In contrast, moderate-to-severe and persistent allergic rhinitis were more common among non-adherent adolescents (*p* = 0.003 and *p* = 0.006, respectively).

Parental health literacy was significantly associated with adolescents’ treatment adherence. Adequate and excellent health literacy were more common among parents of treatment-adherent adolescents, whereas inadequate health literacy was more frequently observed among parents of non-adherent adolescents (*p* < 0.001) (Table 3).

Treatment-adherent adolescents had greater improvements in PSQ, TNSS, and VAS scores at both follow-up assessments. The between-group effect sizes were particularly large for TNSS and VAS, whereas the corresponding associations for PSQ were more modest (Table 4).

### 3.4. Parental Health Literacy and Clinical Outcomes

TNSS and VAS improvement scores differed significantly across the four parental health literacy categories at both T1 and T2 (all Kruskal–Wallis tests, *p* < 0.001).

Moderate positive correlations were observed between parental health literacy and improvement in TNSS (T1: ρ = 0.505; T2: ρ = 0.447; both *p* < 0.001). Similar correlations were found for VAS improvement (T1: ρ = 0.526; T2: ρ = 0.437; both *p* < 0.001).

The associations between parental health literacy and PSQ improvement were weaker (T1: ρ = 0.265, *p* = 0.002; T2: ρ = 0.207, *p* = 0.017). Although PSQ improvement at T1 differed significantly across parental health literacy categories (*p* = 0.010), the overall difference was no longer significant at T2 (*p* = 0.077) (Table 5).

### 3.5. Multivariable Analyses

In multivariable linear regression analyses, baseline PSQ score and treatment adherence were independently associated with the T2 PSQ total score (both *p* < 0.001). Likewise, baseline TNSS and treatment adherence were independently associated with the T2 TNSS score, whereas baseline VAS score and treatment adherence were independently associated with the T2 VAS score (all *p* < 0.001). Age, sex, allergic rhinitis severity, treatment step, and parental TSOY-32 categories were not independently associated with any of the three clinical outcomes (Table 6).

In the Firth penalized multivariable logistic regression analysis, parental health literacy categories were significantly associated with treatment adherence overall (*p* < 0.001). Compared with the inadequate health literacy category, adolescents whose parents had problematic/limited, adequate, or excellent health literacy had higher odds of treatment adherence. However, because of the small number of non-adherent participants in the adequate and excellent health literacy categories, the estimated odds ratios were large and accompanied by wide confidence intervals.

Older age was independently associated with a greater likelihood of treatment adherence, whereas a higher treatment step and a higher baseline PSQ score were independently associated with a lower likelihood of adherence. The model showed high apparent discrimination within the study sample, with an AUC of 0.969 (Table 7).

Because some parental health literacy categories contained very few non-adherent participants, the category-specific odds ratios were large and imprecise. In a sensitivity analysis treating the four TSOY-32 categories as a single ordinal term, each one-category increase in parental health literacy was associated with higher odds of treatment adherence (OR = 11.26, 95% CI 4.54–27.90; *p* < 0.001). This finding supported the direction of the primary categorical analysis under a more parsimonious model specification.

## 4. Discussion

In this study, adolescents with allergic rhinitis had higher PSQ total scores, higher snoring, sleepiness, and behavior subscale scores, and a greater prevalence of PSQ-defined high risk than healthy controls. Significant improvements were observed in PSQ, TNSS, and VAS scores during follow-up, with greater improvements among treatment-adherent adolescents. Although parental health literacy was associated with both treatment adherence and clinical improvement in unadjusted analyses, it did not remain independently associated with clinical outcomes in the multivariable linear regression models. In contrast, treatment adherence remained independently associated with PSQ, TNSS, and VAS outcomes at T2 after multivariable adjustment.

The lower PSQ total and subscale scores observed in the control group indicate a greater burden of sleep-related symptoms among adolescents with allergic rhinitis. Nasal inflammation and obstruction are known to increase upper airway resistance, contributing to snoring, restless sleep, and excessive daytime sleepiness. The substantially higher PSQ-defined high-risk prevalence in adolescents with AR further supports the clinical relevance of evaluating sleep-related symptom burden alongside conventional nasal symptoms. However, because the PSQ is a screening instrument rather than a diagnostic test, these findings should not be interpreted as evidence of a higher prevalence of obstructive sleep apnea [1,6,14,15,20,21].

Significant improvements were observed in both nasal symptoms and sleep-related symptoms throughout the follow-up period. Repeated assessments at three time points allowed us to characterize the temporal pattern of clinical improvement. However, because this was an observational study and no standardized treatment protocol was implemented, the observed improvements should not be interpreted as the direct effect of any specific therapeutic intervention [7,8,9,22,23,24,25,26].

Treatment adherence was associated with greater improvement in both nasal and sleep-related outcomes. Adolescents who adhered to treatment demonstrated greater improvements in PSQ, TNSS, and VAS scores at both early and late follow-up. The association between treatment adherence and T2 clinical outcomes remained significant after adjustment for baseline clinical severity and other potential confounders. Nevertheless, the magnitude of these associations—particularly for TNSS and VAS—requires cautious interpretation. The physician responsible for the final adherence classification had access to TNSS and VAS findings, and improvement in these nasal symptom measures may therefore have influenced the clinician’s judgment of adherence. This creates the possibility of outcome-informed observer and classification bias and may have contributed to the unusually large between-group effect sizes. In contrast, the physician was blinded to PSQ results, making direct outcome-informed classification less likely for PSQ-related associations, although residual adherence misclassification remains possible [8,9,27,28].

Moderate positive correlations were observed between parental health literacy and improvements in TNSS and VAS scores, whereas the associations with PSQ improvement were weaker. Although PSQ improvement at T1 differed across parental health literacy categories, the overall difference was no longer significant at T2. A weak positive ordinal association with PSQ improvement at T2 nevertheless remained evident. These unadjusted findings should therefore be interpreted alongside the multivariable analyses, in which parental health literacy was no longer independently associated with T2 PSQ, TNSS, or VAS outcomes after adjustment for treatment adherence and other covariates [10,11,12,13,20,29,30].

Parental health literacy was strongly associated with treatment adherence in both the primary Firth model and the ordinal sensitivity analysis. However, sparse category-specific data resulted in very large odds ratios with wide confidence intervals. The ordinal analysis supported the direction of the association but not the precise magnitude of the category-specific estimates. Accordingly, these findings should be interpreted primarily in terms of the consistency and direction of the association rather than its exact effect size [10,11,12,13,20,29,30].

When treatment adherence was included in the multivariable models, the associations between parental health literacy and T2 PSQ, TNSS, and VAS outcomes were attenuated and were no longer statistically significant. This pattern is compatible with a possible relationship among parental health literacy, treatment adherence, and clinical outcomes but does not establish mediation. Formal mediation analysis was not performed, and appropriately designed prospective studies are required to evaluate this potential pathway.

Medication-taking behavior and attendance at scheduled follow-up visits were incorporated into a single composite adherence measure and could not be analyzed separately. Because visit attendance may also reflect healthcare access, caregiver organization, socioeconomic circumstances, and engagement with healthcare services, this composite definition may have contributed to the observed association between parental health literacy and adherence.

The similar distribution of parental health literacy categories between the allergic rhinitis and healthy control groups suggests that parental health literacy may be more closely related to day-to-day disease management than to the presence of allergic rhinitis itself. However, the study design was not intended to evaluate the role of parental health literacy in the development of allergic rhinitis, and no conclusions should be drawn regarding disease susceptibility.

These findings may have practical implications for clinical communication in adolescents with AR. Identifying families who experience difficulty understanding or implementing treatment recommendations may help clinicians recognize those who could benefit from additional communication or educational support. Approaches such as simplified written instructions, demonstration of medication administration techniques, and confirmation of understanding using teach-back methods may be reasonable components of routine care. However, no health-literacy or adherence-enhancing intervention was evaluated in the present study; therefore, our findings do not demonstrate that these strategies improve treatment adherence or clinical outcomes [23,24,31,32].

A distinctive feature of the present study is the simultaneous longitudinal evaluation of parental health literacy, treatment adherence, nasal symptom burden, and sleep-related symptoms within the same adolescent AR cohort. Rather than examining these domains in isolation, the study integrated family-level health literacy and treatment-related behavior with repeated clinical assessments at baseline, week 4, and week 8. The contribution of the study therefore lies in characterizing their concurrent associations with repeated nasal and sleep-related outcomes in adolescents with isolated AR. These findings should be considered hypothesis-generating and may inform future prospective studies incorporating objective adherence measures and designs capable of evaluating temporal and mediating relationships.

## 5. Limitations

This study has several limitations. First, because it was conducted at a single center, the generalizability of the findings to populations with different sociodemographic characteristics and healthcare settings may be limited.

Second, treatment adherence was not assessed using an objective or validated adherence measure. The final classification was based on adolescent and parent reports together with clinician judgment, without electronic medication monitoring, pill counts, prescription-refill data, or a prespecified quantitative adherence threshold. This approach may have introduced recall, social desirability, and misclassification biases.

Third, adherence was reduced to a single binary classification for the entire 8-week follow-up period. Separate adherence classifications were not available at T1 and T2, preventing assessment of temporal variation or time-specific adherence patterns.

Fourth, reported medication-taking behavior and attendance at scheduled follow-up visits were incorporated into the same composite adherence construct and were not retained as separate variables. Consequently, medication-specific adherence could not be analyzed independently, and a sensitivity analysis excluding visit attendance was not feasible. Because follow-up attendance may also reflect healthcare access, caregiver organization, socioeconomic circumstances, and engagement with healthcare services, its inclusion may have influenced the observed association between parental health literacy and adherence.

Fifth, the clinician responsible for the final adherence classification was not blinded to TNSS and VAS findings. Clinical improvement in nasal symptoms may therefore have influenced adherence classification, introducing potential observer and classification bias and possibly inflating the associations between adherence and TNSS or VAS outcomes. The clinician was blinded to PSQ results, which reduces this specific source of outcome-informed classification for PSQ-related analyses, although residual adherence misclassification cannot be excluded.

Sixth, both parental health literacy and important components of adherence assessment relied on information provided by participants or caregivers, creating the possibility of common-source and reporting bias.

Seventh, parental health literacy was available in the analytical dataset as the predefined four-level TSOY-32 classification rather than as the original continuous score. Consequently, health literacy was analyzed categorically, which may have resulted in some loss of information. In addition, parental educational level, socioeconomic status, healthcare access, caregiver organization, family responsibilities, and the distribution of caregiving roles were not comprehensively measured or adjusted for, leaving the possibility of residual confounding.

Eighth, sleep-related symptoms were assessed exclusively using the PSQ, and polysomnography was not performed. Consequently, the study evaluated only the risk of sleep-related breathing disorders rather than establishing a diagnosis of obstructive sleep apnea. Furthermore, variables that may influence PSQ scores, including adenoid or tonsillar hypertrophy, obesity, and craniofacial characteristics, were not included in the regression models, leaving the possibility of residual confounding [6,14,15,16,21,33].

Ninth, the number of non-adherent participants was very small in some parental health literacy categories, resulting in sparse cells and potential near-separation. Although Firth penalized regression was used to reduce small-sample and separation-related bias, the category-specific odds ratios remained extremely large and were accompanied by wide confidence intervals. Therefore, the direction of these associations is more interpretable than their exact magnitude.

Tenth, no formal mediation analysis was performed. Accordingly, attenuation of the association between parental health literacy and clinical outcomes after inclusion of treatment adherence in multivariable models should not be interpreted as evidence that adherence mediated this relationship.

Finally, model discrimination was evaluated in the same sample used to fit the Firth regression model, without internal resampling validation or external validation in an independent cohort. Therefore, the reported AUC represents apparent, sample-specific discrimination and should not be interpreted as validated predictive performance.

## 6. Strengths

The principal strength of this study is the integrated longitudinal assessment of parental health literacy, treatment adherence, and repeated nasal and sleep-related outcomes in adolescents with isolated AR. The use of three assessment time points allowed characterization of changes in both conventional nasal symptoms and sleep-related symptoms within the same clinical framework. In addition, simultaneous evaluation of family-level health literacy, treatment-related behavior, and repeated clinical outcomes provides a broader view of factors associated with AR management than assessment of these domains separately.

## 7. Conclusions

Adolescents with allergic rhinitis had a greater burden of sleep-related symptoms than healthy controls. Higher treatment adherence was associated with more favorable nasal and sleep-related outcomes during follow-up, and among the covariates included in the adjusted models, treatment adherence showed the most consistent association with T2 PSQ, TNSS, and VAS outcomes.

Parental health literacy was associated with treatment adherence but was not independently associated with T2 clinical outcomes after multivariable adjustment. Whether treatment adherence represents a pathway linking parental health literacy to clinical outcomes cannot be determined from the present observational study and requires appropriately designed prospective and formal mediation analyses.

Future multicenter studies incorporating objective and time-specific measures of adherence, more comprehensive assessment of family-level confounders, and designs capable of evaluating temporal and mediating relationships are warranted.

## Figures and Tables

**Table 1:** Comparison of Baseline Demographic and Clinical Characteristics Between Adolescents With Allergic Rhinitis and Healthy Controls.

Variable	Allergic Rhinitis (*n* = 132)	Controls (*n* = 120)	*p*
Age (years)	15.00 (13.00–16.00)	15.00 (14.00–16.00)	0.514
Male sex, *n* (%)	64 (48.5)	59 (49.2)	0.914
Family history of atopy, *n* (%)	51 (38.6)	24 (20.0)	0.001
PSQ total score	0.32 (0.00–0.42)	0.00 (0.00–0.00)	<0.001
PSQ snoring subscale	0.50 (0.00–0.75)	0.00 (0.00–0.00)	<0.001
PSQ sleepiness subscale	0.25 (0.00–0.25)	0.00 (0.00–0.00)	<0.001
PSQ behavior subscale	0.00 (0.00–0.20)	0.00 (0.00–0.00)	<0.001
PSQ-defined high risk (≥0.33), *n* (%)	66 (50.0)	6 (5.0)	<0.001
Parental TSOY-32 categories, *n* (%)			0.754
Inadequate	26 (19.7)	30 (25.0)	
Problematic/Limited	37 (28.0)	34 (28.3)	
Adequate	48 (36.4)	39 (32.5)	
Excellent	21 (15.9)	17 (14.2)	

Data are presented as median (interquartile range [IQR]) or *n* (%). Continuous variables were compared using the Mann–Whitney *U* test, and categorical variables using the Pearson χ^2^ test or Fisher’s exact test when appropriate. The TSOY-32 was completed by a parent or primary caregiver for each adolescent. PSQ, Pediatric Sleep Questionnaire; TSOY-32, Turkish Health Literacy Scale-32.

**Table 2:** Changes in Clinical Outcomes Between Baseline (T0), Week 4 (T1), and Week 8 (T2) in Adolescents With Allergic Rhinitis.

Variable	T0	T1	T2	Friedman χ^2^	*p*
PSQ total score	0.32 (0.00–0.42)	0.12 (0.00–0.35)	0.00 (0.00–0.17)	120.74	<0.001
PSQ snoring subscale	0.50 (0.00–0.75)	0.25 (0.00–0.50)	0.00 (0.00–0.25)	114.36	<0.001
PSQ sleepiness subscale	0.25 (0.00–0.25)	0.00 (0.00–0.25)	0.00 (0.00–0.05)	51.49	<0.001
PSQ behavior subscale	0.00 (0.00–0.20)	0.00 (0.00–0.20)	0.00 (0.00–0.00)	54.51	<0.001
TNSS	7.00 (5.00–9.00)	3.00 (2.00–6.00)	3.00 (2.00–6.00)	172.57	<0.001
VAS	6.00 (5.00–8.00)	3.00 (2.00–5.00)	3.00 (2.00–5.00)	148.59	<0.001

Data are presented as median (IQR). Overall changes across T0, T1, and T2 were analyzed using the Friedman test. For variables with significant Friedman test results, pairwise comparisons were performed using the Bonferroni-adjusted Wilcoxon signed-rank test. Bonferroni-adjusted pairwise comparisons were as follows: PSQ total score, T0–T1 *p* < 0.001, T0–T2 *p* < 0.001, and T1–T2 *p* < 0.001; TNSS, T0–T1 *p* < 0.001, T0–T2 *p* < 0.001, and T1–T2 *p* = 0.011; VAS, T0–T1 *p* < 0.001, T0–T2 *p* < 0.001, and T1–T2 *p* = 0.805. The proportion of participants classified as high risk according to the PSQ was 50.0% (*n* = 66) at T0, 28.0% (*n* = 37) at T1, and 19.7% (*n* = 26) at T2. Comparisons between T0–T1 and T0–T2 were performed using the Bonferroni-adjusted McNemar test, with adjusted *p* < 0.001 for both comparisons. PSQ, Pediatric Sleep Questionnaire; TNSS, Total Nasal Symptom Score; VAS, Visual Analog Scale.

**Table 3:** Comparison of Baseline Demographic and Clinical Characteristics According to Treatment Adherence.

Variable	Adherent (*n* = 90)	Non-Adherent (*n* = 42)	*p*
Age (years)	15.00 (14.00–16.00)	14.00 (13.00–15.75)	0.041
Male sex, *n* (%)	46 (51.1)	18 (42.9)	0.377
Family history of atopy, *n* (%)	38 (42.2)	13 (31.0)	0.216
Baseline PSQ total score	0.15 (0.00–0.35)	0.40 (0.35–0.50)	<0.001
Baseline TNSS	6.00 (4.25–8.00)	9.00 (6.00–10.75)	<0.001
Baseline VAS	5.50 (4.00–7.75)	8.00 (6.00–9.00)	0.001
Treatment step	2.00 (1.00–3.00)	3.00 (2.00–3.00)	<0.001
Moderate-to-severe allergic rhinitis, *n* (%)	35 (38.9)	28 (66.7)	0.003
Persistent allergic rhinitis, *n* (%)	37 (41.1)	28 (66.7)	0.006
Parental TSOY-32 categories, *n* (%)			<0.001
Inadequate	**3 (3.3)**	**23 (54.8)**	
Problematic/Limited	**22 (24.4)**	**15 (35.7)**	
Adequate	**47 (52.2)**	**1 (2.4)**	
Excellent	**18 (20.0)**	**3 (7.1)**	

Data are presented as median (IQR) or *n* (%). Continuous variables were compared using the Mann–Whitney *U* test, and categorical variables using the Pearson χ^2^ test or Fisher’s exact test, as appropriate. Treatment adherence represents a single clinician-assessed composite classification covering the entire 8-week follow-up period and incorporating reported medication-taking behavior together with attendance at scheduled follow-up visits. The TSOY-32 was completed by the adolescent’s parent or primary caregiver. PSQ, Pediatric Sleep Questionnaire; TNSS, Total Nasal Symptom Score; VAS, Visual Analog Scale; TSOY-32, Turkish Health Literacy Scale-32.

**Table 4:** Comparison of Clinical Improvement According to Treatment Adherence.

Outcome	Adherent (*n* = 90)	Non-Adherent (*n* = 42)	*p*	Rank-Biserial *r*
PSQ improvement, T1	0.08 (0.00–0.20)	0.00 (0.00–0.02)	<0.001	0.409
PSQ improvement, T2	0.15 (0.00–0.35)	0.00 (0.00–0.20)	0.016	0.253
TNSS improvement, T1	4.00 (3.00–5.00)	0.00 (0.00–1.00)	<0.001	0.929
TNSS improvement, T2	4.00 (3.00–5.00)	1.00 (0.00–1.75)	<0.001	0.810
VAS improvement, T1	4.00 (3.00–4.00)	0.00 (0.00–1.00)	<0.001	0.890
VAS improvement, T2	4.00 (3.00–4.00)	1.00 (0.00–2.00)	<0.001	0.716

Data are presented as median (IQR). Groups were compared using the Mann–Whitney *U* test. Improvement scores were calculated as the difference between baseline and follow-up values (T0−T1 or T0−T2), with positive values indicating greater clinical improvement. Rank-biserial correlation coefficients are presented as measures of effect size. PSQ, Pediatric Sleep Questionnaire; TNSS, Total Nasal Symptom Score; VAS, Visual Analog Scale.

**Table 5:** Comparison of Clinical Improvement According to Parental Health Literacy.

Outcome	Inadequate (*n* = 26)	Problematic/Limited (*n* = 37)	Adequate (*n* = 48)	Excellent (*n* = 21)	Kruskal–Wallis *p)*	Spearman ρ	Spearman *p*
PSQ improvement, T1	0.00 (0.00–0.09)	0.00 (0.00–0.08)	0.08 (0.00–0.20)	0.08 (0.00–0.30)	0.010	0.265	0.002
PSQ improvement, T2	0.00 (0.00–0.27)	0.05 (0.00–0.18)	0.17 (0.00–0.35)	0.08 (0.00–0.40)	0.077	0.207	0.017
TNSS improvement, T1	1.00 (0.00–1.00)	2.00 (0.00–4.00)	3.00 (2.00–4.25)	4.00 (3.00–6.00)	<0.001	0.505	<0.001
TNSS improvement, T2	1.00 (0.00–2.75)	2.00 (1.00–4.00)	3.00 (2.75–5.00)	4.00 (3.00–6.00)	<0.001	0.447	<0.001
VAS improvement, T1	0.00 (0.00–1.00)	2.00 (0.00–4.00)	4.00 (3.00–4.00)	4.00 (3.00–5.00)	<0.001	0.526	<0.001
VAS improvement, T2	1.00 (0.00–2.75)	2.00 (1.00–4.00)	4.00 (3.00–4.00)	3.00 (3.00–5.00)	<0.001	0.437	<0.001

Data are presented as median (IQR). Parental health literacy categories were determined using the Turkish Health Literacy Scale-32 (TSOY-32), completed by the adolescent’s parent or primary caregiver. Overall comparisons among the four health literacy categories were performed using the Kruskal–Wallis test. The ordinal association between parental health literacy and clinical improvement was assessed using Spearman’s rank correlation analysis. Improvement scores were calculated as T0−T1 or T0−T2, with positive values indicating greater clinical improvement. PSQ, Pediatric Sleep Questionnaire; TNSS, Total Nasal Symptom Score; VAS, Visual Analog Scale; TSOY-32, Turkish Health Literacy Scale-32.

**Table 6:** Factors Associated With Clinical Outcomes at the Second Follow-up (T2): Multivariable Linear Regression Analyses.

Variable	T2 PSQ B (95% CI)	*p*	T2 TNSS B (95% CI)	*p*	T2 VAS B (95% CI)	*p*
Baseline score	0.212 (0.091 to 0.332)	<0.001	0.536 (0.349 to 0.722)	<0.001	0.294 (0.130 to 0.459)	<0.001
Treatment adherence	−0.235 (−0.308 to −0.162)	<0.001	−4.105 (−4.869 to −3.342)	<0.001	−3.425 (−4.108 to −2.741)	<0.001
Treatment step	0.016 (−0.027 to 0.058)	0.466	−0.103 (−0.625 to 0.419)	0.699	0.182 (−0.265 to 0.630)	0.425
Age	−0.006 (−0.018 to 0.006)	0.307	0.037 (−0.090 to 0.164)	0.571	0.050 (−0.072 to 0.172)	0.417
Male sex	−0.001 (−0.039 to 0.038)	0.972	0.305 (−0.161 to 0.771)	0.199	0.132 (−0.314 to 0.577)	0.562
Moderate-to-severe AR	0.005 (−0.063 to 0.074)	0.878	0.312 (−0.608 to 1.233)	0.506	0.689 (−0.190 to 1.568)	0.125
TSOY-32 Category 2 vs. 1	0.054 (−0.027 to 0.135)	0.190	0.504 (−0.466 to 1.474)	0.308	0.521 (−0.385 to 1.427)	0.259
TSOY-32 Category 3 vs. 1	0.039 (−0.037 to 0.115)	0.312	0.647 (−0.316 to 1.610)	0.188	0.349 (−0.642 to 1.339)	0.491
TSOY-32 Category 4 vs. 1	0.002 (−0.080 to 0.084)	0.955	0.021 (−0.950 to 0.993)	0.966	0.099 (−0.945 to 1.142)	0.853
R^2^	0.699		0.832		0.764	
Adjusted R^2^	0.677		0.820		0.747	
Model *p*	<0.001		<0.001		<0.001	

Data are presented as unstandardized regression coefficients (B), 95% confidence intervals (CIs), and *p* values. Separate multivariable linear regression models were constructed for PSQ, TNSS, and VAS scores at the second follow-up (T2). Each model included the corresponding baseline score, treatment adherence, treatment step, age, sex, allergic rhinitis severity, and parental health literacy category. For parental TSOY-32 categories, inadequate health literacy served as the reference category. For treatment adherence, the non-adherent group was the reference category; for sex, female sex was the reference category; and for disease severity, mild allergic rhinitis was the reference category. PSQ, Pediatric Sleep Questionnaire; TNSS, Total Nasal Symptom Score; VAS, Visual Analog Scale; TSOY-32, Turkish Health Literacy Scale-32; CI, confidence interval.

**Table 7:** Factors Associated With Treatment Adherence Among Adolescents With Allergic Rhinitis: Firth Penalized Multivariable Logistic Regression Analysis.

Variable	OR	95% CI	*p*
Parental TSOY-32 category			<0.001 *
Problematic/Limited vs. Inadequate	27.730	4.014–191.540	<0.001
Adequate vs. Inadequate	810.760	56.577–11,618.457	<0.001
Excellent vs. Inadequate	420.733	26.384–6709.248	<0.001
Treatment step (per one-step increase)	0.306	0.118–0.794	0.015
Age (per one-year increase)	1.968	1.252–3.093	0.003
Baseline PSQ total score (per 0.10-point increase)	0.639	0.437–0.936	0.021
Model AUC	0.969	—	—

The dependent variable was treatment adherence, with treatment-adherent adolescents defined as the outcome category. Parental TSOY-32 categories were entered as nominal categorical variables, with inadequate health literacy as the reference category. Firth penalized logistic regression was used to reduce bias associated with sparse data and potential separation. Because the baseline PSQ total score ranges from 0 to 1, odds ratios are reported for each 0.10-point increase. The area under the receiver operating characteristic curve (AUC) was calculated using the predicted probabilities generated from the Firth penalized logistic regression model. Because these probabilities were generated in the same dataset used to fit the model and no internal or external validation was performed, the AUC represents apparent, in-sample discrimination rather than validated predictive performance. OR, odds ratio; CI, confidence interval; AUC, area under the receiver operating characteristic curve; PSQ, Pediatric Sleep Questionnaire; TSOY-32, Turkish Health Literacy Scale-32. * The overall contribution of parental TSOY-32 categories to the model was evaluated using the penalized likelihood ratio test.

## Data Availability

The original contributions presented in this study are included in the article. Further inquiries can be directed to the corresponding author.
